# A no film slot blot for the detection of developing *P*. *falciparum* oocysts in mosquitoes

**DOI:** 10.1371/journal.pone.0174229

**Published:** 2017-04-21

**Authors:** Bryan Grabias, Nitin Verma, Hong Zheng, Abhai K. Tripathi, Godfree Mlambo, Merribeth J. Morin, Emily Locke, Sanjai Kumar

**Affiliations:** 1 Laboratory of Emerging Pathogens, Division of Emerging and Transfusion Transmitted Diseases, Center for Biologics Evaluation and Research, Food and Drug Administration, Silver Spring, Maryland, United States of America; 2 The Johns Hopkins Malaria Research Institute, Bloomberg School of Public Health, The Johns Hopkins University, Baltimore, Maryland, United States of America; 3 Department of Molecular Microbiology and Immunology, Bloomberg School of Public Health, The Johns Hopkins University, Baltimore, Maryland, United States of America; 4 PATH Malaria Vaccine Initiative, Washington DC, United States of America; Université Pierre et Marie Curie, FRANCE

## Abstract

Non-microscopy-based assays for sensitive and rapid detection of *Plasmodium* infection in mosquitoes are needed to allow rapid and high throughput measurement of transmission intensity and malaria control program effectiveness. Here, we report on a modified enhanced chemiluminescence-based slot blot assay for detection of *Plasmodium falciparum* (*Pf*) circumsporozite protein (*Pf*CSP) expressed on parasite oocysts developing inside the mosquito midgut. This modified assay has several novel features that include eliminating the need for exposure to autoradiography (AR) film, as well as utilizing a novel high affinity anti-CSP antibody, and optimizing assay procedures resulting in significant reduction in the time required to perform the assay. The chemiluminescent signal for the detection of *Pf*CSP in mosquito samples was captured digitally utilizing the C-Digit blot scanner that, allowed the detection of 0.01 pg of recombinant *P*. *falciparum* CSP and as few as 0.02 *P*. *falciparum* oocysts in a little over two hours. The earlier ECL-SB detected rCSP and oocysts and took approximately 5 h to perform. Whole mosquito lysates from both high and low prevalence—infected mosquito populations were prepared and evaluated for *Pf*CSP detection on the ECL-SB by both AR film and digital data capture and analysis. There was a 100% agreement between the AR film and the C-Digit scanner methods for *Pf*CSP detection in randomly sampled mosquitoes. This novel “No Film” Slot Blot assay obviates the need for AR film exposure and development and significantly reduces the assay time enabling widespread use in field settings.

## Introduction

There are still no available vaccines against several important infectious diseases including malaria. A major hurdle to developing novel vaccines is that many assays currently used for antigen discovery and characterization are particularly cumbersome and not amenable to high throughput analysis of many candidates at once. There is an urgent need for highly sensitive assays that could be applied in high throughput formats to evaluate pre-clinical and clinical efficacy in surveillance and epidemiological studies.

Malaria remains a pressing global health issue, causing an estimated 438,000 deaths and an estimated 214 million new infections worldwide in 2015 [1]. A critical strategy for combating the spread of malaria-causing parasites, thus facilitating elimination and eradication, relies upon blocking mosquito-borne transmission by increasing vector resistance to parasite development through either vaccines, drugs, or genetic manipulation. Both naturally acquired [2–4] and vaccination-induced antibodies [5–8] can interrupt parasite maturation in mosquitoes by preventing fertilization of male and female gametes, interfering with zygote formation, or blocking the ookinete to midgut oocyst transition where primordial sporozoites are formed before migrating to the salivary glands. Currently, the standard method of measuring the efficacy of transmission-blocking interventions is an *ex vivo* assay, the standard membrane feeding assay (SMFA). In this assay, mosquitoes are fed an infectious blood meal containing transmission-blocking antibodies and, after approximately 8 days, individual developing oocysts in the mosquito midgut are stained and enumerated via microscopy [9, 10]. Dissection and accurate assessment of mosquito midguts is a labor-intensive process that is not particularly well-suited to the processing of the hundreds or thousands of samples that are generated in large vaccine trials or epidemiological studies. Additionally, counts obtained from this method typically exhibit a high degree of variance and are potentially prone to error [10, 11]. The enhanced chemiluminescent slot blot (ECL-SB) assay was developed in our laboratory to provide an alternative to microscopy for the immunological detection of *P*. *falciparum* oocysts in infected mosquito populations. Utilizing the monoclonal antibody 2A10 specific to the repeat NANP unit of *P*. *falciparum* circumsporozoite protein (CSP), the ECL-SB demonstrated high sensitivity and specificity, detecting as little as 1.25 pg of recombinant CSP protein and as few as 0.25 oocyst from whole mosquito lysates [12].

Other methods for the evaluation of transmission-blocking treatments have been developed and include detection of *P*. *falciparum* antigen via an ELISA [13, 14] or amplification of parasite nucleic acids with PCR-based assays [15, 16]. Standard calorimetric ELISAs that rely on the quantitation of a colored product can exhibit sensitivities of anywhere from 12.5–250 *P*. *falciparum* sporozoites [14, 17–19]. In one study, the sensitivities of the ECL-SB and a colorimetric CSP-ELISA for the detection of *Pf* oocysts in mosquito midguts at 10 days post-infectious feed were determined to be comparable, while the ECL-SB displayed superior detection at earlier time points [20]. RT-PCR has displayed sensitivities of 1.5–75 sporozoites [16, 21]. Our laboratory also recently developed a chemiluminescent ELISA for the detection of *P*. *falciparum* in whole mosquito lysates. Our data demonstrated that the ECL ELISA could detect as few as 5 *Pf* sporozoites or 0.056 day 8 *Pf* oocysts [22].

To be considered as part of a sustainable testing strategy in field settings, mosquito screening tests should be relatively streamlined, requiring little processing and minimal technological infrastructure. The slot blot assay was originally designed as a field-applicable screening assay for *Plasmodium*-infected mosquito specimens. The slot blot employs a commercially available acrylic plate attached to a vacuum line to transfer sample proteins onto a nitrocellulose membrane. In contrast to other typical screening assays, mosquitoes or other tissues can be homogenized in lysis buffer and directly applied to the ECL-SB membrane obviating the need for excessive sample processing. After several wash steps and antibody incubations, similar to the processing of a Western blot, the membrane can be exposed to autoradiography film and individual band intensities can be extracted and analyzed from digitally scanned film. Originally, the slot blot required approximately five hours to perform. This significant time investment presents a potential logistical hurdle for field sites, especially at higher sample throughputs. Additionally, the availability of a dark room and film processing machine cannot be guaranteed in resource limited field settings. Data collection via exposure to autoradiography film also represents an inherent cost burden as funds must be continually spent to ensure a constant supply of fresh reagents for developing film. Herein, we report on recent advancements and modifications to our previously published ECL-SB protocol to enhance assay sensitivity and reduce cost and technological or logistical barriers that would prevent its widespread adoption as the premier field assay for the detection of *P*. *falciparum* antigens in whole mosquito specimens.

Firstly, we have reduced the use of vacuum suction for slot blot sample application and membrane washes. Elimination of vacuum-assisted wash steps not only reduces technological requirements for processing the ECL-SBs but also reduced overall assay times significantly. To further shorten the assay run time, we have reduced the overall number of wash steps required to process ECL-SBs and the duration of the membrane blocking step. To compensate for these changes, which could potentially impact assay sensitivity and specificity, all wash steps are now performed with blocking buffer instead of PBS as originally described [12].

Our laboratory also developed a novel monoclonal antibody specific to the immunodominant NANP regions of *Pf*CSP to maximize assay sensitivity and to further enhance the performance of the modified ECL-SB assay. We characterized the sensitivity and specificity of this new antibody (C3103) when compared to the mAb 2A10 clone used previously in the slot blot assay. Importantly, whereas the initial iteration of the slot blot assay employed a sandwich immunoassay format to generate a chemiluminescent reporter signal, the revised slot blot protocol now employs a single AP-conjugated anti-CSP antibody that simultaneously detects *P*. *falciparum* antigen and generates a luminescent response for detection and quantitation. Finally, to eliminate the need for film development and any associated burdens, we have characterized the performance of the ECL-SB when data is acquired via a digitally scanned image obtained using the LI-COR C-Digit Blot Scanner (C-Digit).

We find that the new ECL-SB protocol results in significantly improved sensitivity and specificity while simultaneously simplifying sample and blot processing. Thus, the slot blot assay is amenable to widespread use in field sites for the detection of *P*. *falciparum* parasites in mosquito vectors. The performance characteristics of the ECL-SB establish its value as a potential replacement/supplement for a Western blot, or other similar immunological assays in preclinical and clinical vaccine development processes for applications such as antigen discovery, assessment of immune responses or quantification of antigenic component(s) in a vaccine formulation.

## Materials & methods

### rPfCSP

Recombinant *P*. *falciparum* T4 CSP (r*Pf*CSP) with the amino acid sequence 27–123[NANPNVDP]_3_[NANP]_21_300–411 (GB Accession: AAA29555.1) was expressed in *E*. *coli* and purified on a heparin sepharose affinity column. Recombinant expression, purification, and antigenic characterization of r*Pf*CSP are described elsewhere [23, 24]. Quantitation of total protein and the creation of serial dilutions was performed as previously reported [12].

### mAb 2A10

Anti-*Pf*CSP mAb 2A10 was produced in a hybridoma cell line obtained from the MR4/ATCC, Virginia [25]. Ascites production in mice and antibody purification using Protein G affinity chromatography was accomplished using a commercial source (Harlan Laboratories Inc. Madison, WI). mAb 2A10 was characterized for its immune-reactivity in IFA using *P*. *falciparum* sporozoites and in ELISA and Western blot using r*Pf*CSP.

### Generation of novel monoclonal anti-CSP antibody C3103

Six to eight week old female BALB/c inbred mice were purchased from NIH. All mice used in this study were maintained under appropriate conditions at the Center for Biologics Evaluation and Research, Silver Spring, MD. This study was done in accordance with the guidelines for the care and use of laboratory animals specified by the National Institutes of Health. This protocol was approved by the Institutional Animal Care and Use Committee of the Center for Biologics Evaluation and Research under Animal Study Protocol 2014–06. To produce anti-PfCSP monoclonal antibodies, BALB/c mice were immunized with two doses of 10^5^ *P*. *falciparum* 3D7 sporozoites in complete RPMI medium by intravenous route followed by an additional two doses of 5x10^4^ *Pf*3D7 sporozoites after two weeks. Three weeks later, a final booster dose of 20 μg of purified recombinant *Pf*CSP protein was administered intravenously. Three days after the final dose, mice were sacrificed by cervical dislocation with considerable efforts made to minimize suffering. Spleens were harvested and fused with Sp2/0-Ag14 mouse myeloma cells in the presence of polyethylene glycol 1500 (50% W/V) (Roche, Germany) as described by Kohler and Milstein [26]. Positive hybridomas secreting anti-r*Pf*CSP antibodies were screened in ELISA against immobilized r*Pf*CSP. A secondary screening was performed to identify the hybridoma secreting antibodies specific for (NANP)_6_ peptide. The wells which were positive in ELISA for both r*Pf*CSP and (NANP)_6_ were single cell cloned using the limiting cell dilution method and the reactivity of anti-*Pf*CSP antibody secreted was tested by ELISA and in an immunofluorescence assay (IFA). The anti-*Pf*CSP mAb clone C3103 was determined to possess both the highest ELISA reactivity against r*Pf*CSP and (NANP)_6_ peptide and the strongest IFA signal against *Pf*SPZ. This clone was subsequently selected for ascites production and the antibody was purified using a rProtein A column on the AKTA purification system and retested both by ELISA and IFA for the end point titer.

### Preparation of anti-PfCSP-alkaline phosphatase conjugate

Covalent linkage of the anti-CSP mAb C3103 or 2A10 with an alkaline phosphatase reporter enzyme was achieved using the Lightning-Link^®^ Alkaline Phosphatase Conjugation Kit according to the manufacturer’s instructions (Innova Biosciences, Cambridge, UK). Briefly, 100 μg of antibody was added to LL-modifier reagent and gently mixed. This antibody-buffer mixture was then directly combined with lyophilized Lightning-Link^®^ reagent and incubated at 4°C overnight. The reaction was finally quenched with LL-quencher reagent and the conjugated antibodies were stored as stock solutions at 4°C.

### Transmission of *P*. *falciparum* in mosquitoes and enumeration of oocysts

Oocyst lysates were prepared as described in our previous report [12]. Briefly, *P*. *falciparum* gametocytes (NF54) prepared from enriching asexual blood stage *P*. *falciparum* parasite cultures were fed to *A*. *stephensi* mosquitoes in a standard membrane feeding assay [27]. Human plasma and red blood cells used for the *P*. *falciparum* cultures were purchased from Interstate Blood Bank, Inc. (Memphis, TN: www.interstatebloodbank.com). Fed mosquitoes were maintained in cages kept at 80% relative humidity and for up to nine days after feeding to allow maturation of developing oocysts. Seven to nine days post infectious blood meal, a random sample of 20 mosquitoes were collected from which midguts were harvested. Only midguts from mosquitoes with eggs at the time of dissection were analyzed. Mosquito midguts were stained with 0.05% mercurochrome solution for 20–45 minutes on a regular microscope slide and the number of *Pf*Oocysts per midgut was enumerated by two independent examiners via microscopy. Average oocyst burden and overall prevalence of *P*. *falciparum* oocysts of the microscopically dissected mosquitoes were then utilized as population estimates for the remaining mosquitoes fed the same infectious blood meal. These mosquitoes were pooled and used to generate *Pf* oocyst lysate.

### PfCSP detection in whole mosquito lysates

The ECL-SB has been optimized for the detection of *P*. *falciparum* parasites in whole mosquito lysates. We compared the sensitivity of the modified slot blot protocol using the C-Digit blot scanner to that of autoradiograph film in the detection of parasites from two independent populations of *A*. *stephensi* mosquitoes. One population was fed an infectious blood meal containing a high concentration of *Pf* gametocytes, while the other population was fed with low gametocytemic blood. A random sample of mosquitoes from each population was harvested 8 days post blood meal and subjected to microscopic dissection for the estimation of population prevalence and oocyst intensity as described above. Each individual uninfected or infected mosquito was placed into a single tube and homogenized with a piston in 50 μL of lysis buffer (1X TBS, 0.5% SDS). The lysates were subsequently vortexed for 20 s and then boiled for 5 minutes. Any particulate matter was pelleted via centrifugation and the supernatant was collected, applied to the slot blot apparatus, and analyzed.

### Performance of ECL slot blot assay

The ECL-SB was carried out as described previously [12] with several modifications to streamline the procedure. Briefly, serial dilutions of r*Pf*CSP (ranging from 1–0.008 pg) and *Pf*Oocyst lysate (ranging from 0.25–0.004 PfOocysts/20 μl) in TBS plus 0.5% SDS were loaded in duplicate in the wells in the ECL-SB apparatus. The protein samples were allowed to bind to nitrocellulose paper for 15 minutes. The membrane was then subjected to 3× 10 minute incubations in iBlock blocking buffer (Applied Biosystems, T2015, Foster City, CA) and probed with AP-conjugated anti-PfCSP mAb 2A10 or C3103. After subsequent 3× 10 minute washes in iBlock blocking buffer, the membrane was rinsed twice for two minutes with 25 ml of 1X assay buffer and then incubated with 6 ml of ECL-substrate solution at RT for five minutes and bands were visualized by either exposure to an AR film and developed (Kodak X-OMAT 1000A) or digitally captured on the C-Digit blot scanner (LI-COR, Lincoln, NE). The ECL-reagents used in this assay were purchased as a kit (Life Technologies,Western-StarTM Immunodetection System, T1046, Grand Island, NY) from Applied Biosystems, (Bedford, MA).

### Data acquisition and analysis

AP reporter signal from ECL-SB membranes was either obtained by extracting band intensities from scanned, exposed film using ImageJ software (http://rsbweb.nih.gov/ij/) or simultaneously scanned and analyzed using the LI-COR C-digit blot scanner. When film was used, the integrated optical density (IOD) of each band was determined by measuring the band intensity in a ‘gated area’. The dimensions of the gated area for IOD determination was kept constant for each band on the scanned image. Data analysis using the C-Digit scanner proceeded in a similar manner utilizing the native scanner software according to the manufacturer’s recommendations.

### Detection of PfCSP

The Michaelis-Menten (M-M) equation [22] was applied to estimate the IOD corresponding to the concentration of rPfCSP using ECL-SB. The M-M equation is a non-linear model which indicated that the IOD approached a saturation level as the amount of rPfCSP reached a specific concentration. This equation is defined as

$$y=\frac{{\text{V}}_{\text{m}}\times x}{x+\text{K}}$$

where V_m_ is the horizontal asymptote of IOD and K is the concentration of r*Pf*CSP (pg) at which the IOD is a half of the horizontal asymptote (K>0). The M-M equation was used to define the relationship between IOD (y) and r*Pf*CSP amount or oocyst count (x).

### Precision

We measured the intra-assay variability (within run) and the inter-assay variability (between runs) as two distinct measurements to determine the ability of the ECL-SB to consistently detect *E*. *coli* expressed r*Pf*CSP and native *Pf*CSP on *Pf*Oocysts. To determine the intra-assay variability, 5 pg of rPfCSP or 4 PfOocysts were run eight times in each experiment. The mean and the standard deviation (SD) of IODs obtained from those eight runs were used for the calculation. The inter-assay variability was calculated by using the means of eight runs obtained on three different days. The assay precision was calculated as percent of coefficient of variation (CV).

$$Intra-assay(CV\%)=\frac{SD}{\text{Mean}}x100$$

where the mean and standard deviation (SD) were calculated among samples in the same experiment, and

$$Inter-assay(CV\%)=\frac{SD_{Means}}{\text{Grand Mean}}x100$$

where the SD of Means and Grand Mean was calculated in experiments conducted on three days.

### Comparison between film and digitally scanned blots for prevalence estimation in vector populations

The strength of the correlation between the overall diagnostic results for both of these two distinct methods of analysis was confirmed using a chi squared test for homogeneity when compared to the original results obtained via microscopy.

## Results & discussion

### Slot blot optimization

**Fig 1:**
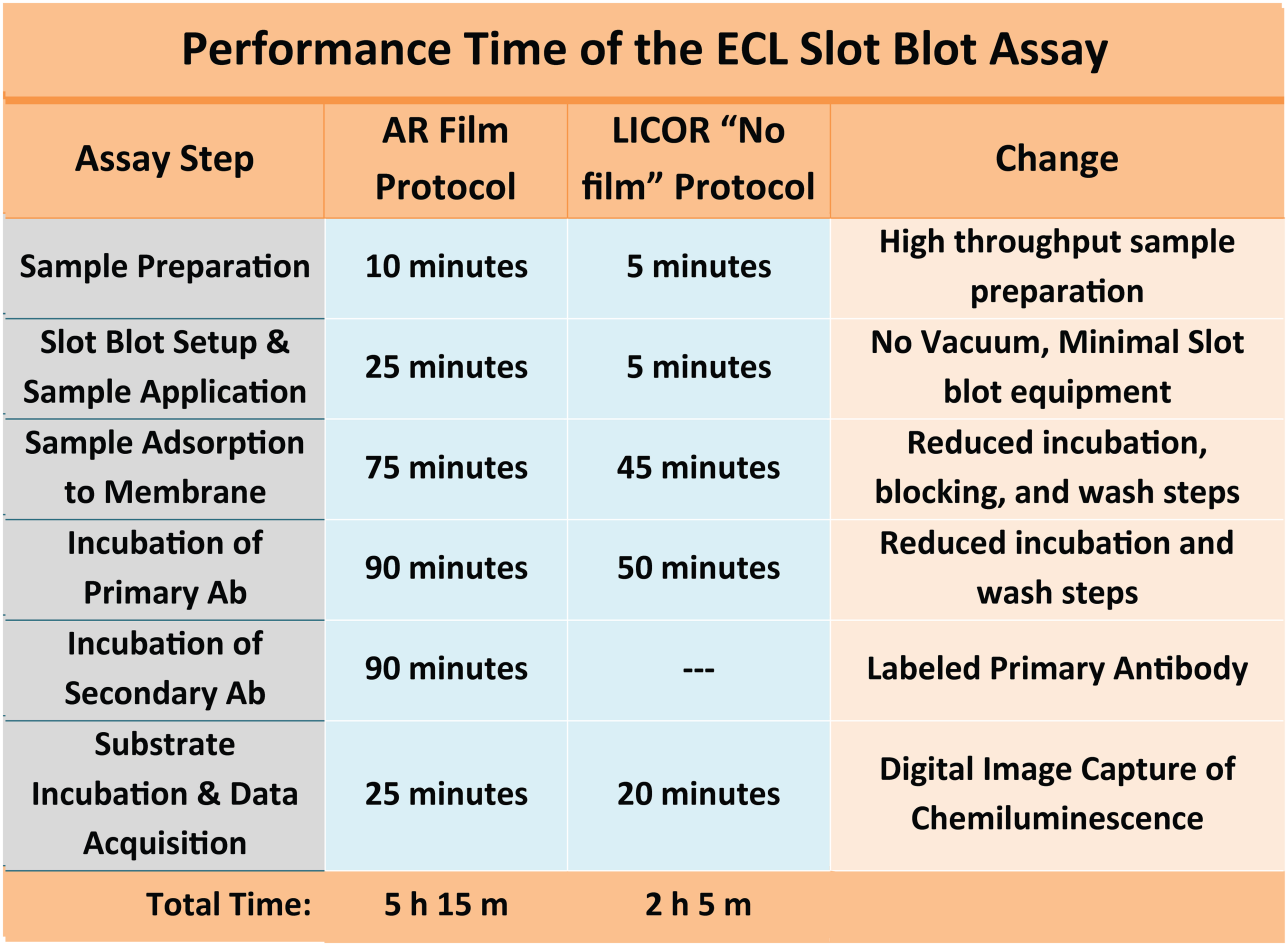
Summary of the modifications to the slot blot assay protocol. A number of changes were implemented to improve overall assay performance, reduce assay time, and minimize reagent and film requirements to better allow broad usage of the ECL-SB in resource poor field sites. The time required to perform the slot blot has been reduced by more than half of the original protocol length and the sensitivity for *Pf*oocysts has been improved from to 0.25 oocyst to 0.02 oocyst.

**Fig 2:**
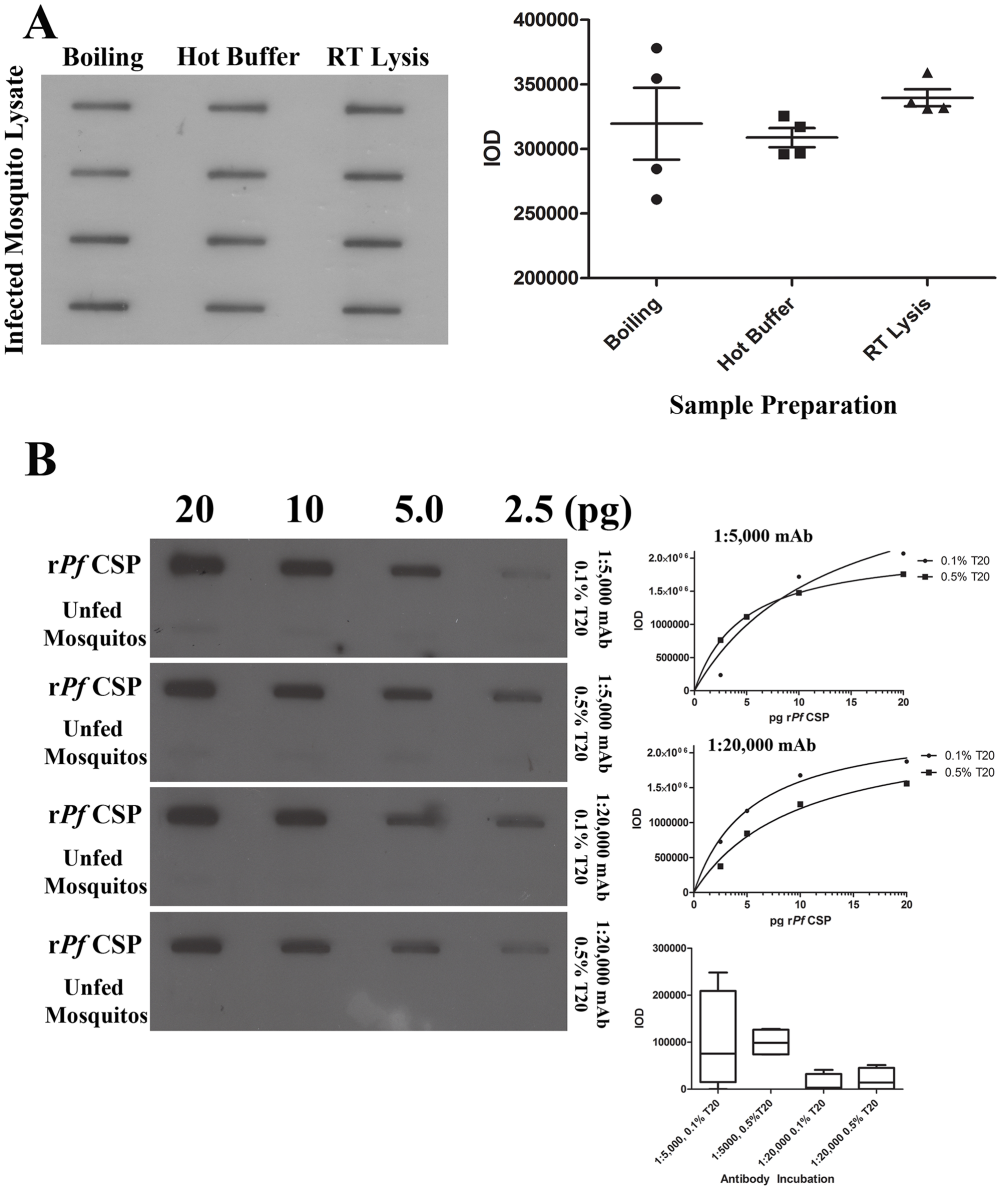
Optimization of sample processing and antibody incubations. Before being applied to the ECL-SB membrane, protein samples were typically boiled in lysis buffer for ten minutes. In order to potentially shorten assay time, we compared band intensities when samples containing 2 oocysts per well were subjected to either the standard boiling protocol (left), homogenized directly in hot (100°C) lysis buffer (middle), or lysed and applied to the slot blot all at room temperature (right) (A). Our data indicate that the RT lysis method is at least as good and statistically not significantly different from boiling samples for ten minutes thus boiling was removed from the protocol. We also evaluated different secondary antibody and detergent concentrations during the secondary antibody incubation step and the overall effects on slot blot band intensities and estimated detection limits of recombinant *Pf*CSP are shown (B). Integrated optical densities were obtained for each specimen tested under each antibody and detergent concentration and fit to a Michaelis-Menten regression curve. Utilizing lysates of uninfected mosquito specimens as negative controls, we established a cutoff threshold of mean IOD + 2 standard deviations to estimate assay sensitivity under each condition being evaluated. We observed that highest sensitivity and signal-to-noise ratio was obtained when a secondary antibody dilution of 1:20,000 was employed with 0.1% Tween-20.

To better streamline the slot blot assay and enhance overall performance, we made a number of general changes to the protocol of the PfCSP slot blot described previously [12] (Fig 1). Our prior slot blot procedure involved boiling whole mosquito homogenates for ten minutes prior to application onto the slot blot membrane. Our studies have determined that no loss of signal occurs if the boiling step is omitted (Fig 2A), with 2 oocysts per well exhibiting similar intensities to whole mosquitoes homogenized in hot (100°C) lysis buffer (p = 0.7) or in lysis buffer at room temperature (p = 0.5). We also observed that vacuum suction was not absolutely required during sample application and the initial membrane wash steps (https://doi.org/10.6084/m9.figshare.4786120.v1). Simultaneous removal of these two steps greatly reduced the time required for binding of samples to the membrane. We also reduced the blocking time by half with no noticeable effect on performance (https://doi.org/10.6084/m9.figshare.4786153.v1).

To better discriminate between *Pf*CSP-specific signal and background, we also evaluated a number of incubation conditions for the secondary antibody. Specifically, utilizing exposure to autoradiography film, we evaluated *Pf*CSP mAb performance at both 1:5,000 and 1:20,000 dilutions in the presence of either 0.1% or 0.5% Tween-20 detergent (T20). As expected, we observed that higher antibody concentrations resulted in higher extracted band intensities (1/5,000: 237294–2068517 arbitrary units (au); 1/20,000: 375712–1875674 au), but primarily for higher concentrations of r*Pf*CSP. In contrast, the lower antibody dilution (1:20,000) combined with the higher detergent concentration exhibited the lowest background signal levels when lysates prepared from unfed mosquitoes that did not receive a *P*. *falciparum* infected blood meal were run as controls (Fig 2B). The background-subtracted integrated optical density values were plotted and fit to a Michaelis-Menten best-fit curve. The limits of detection were determined for each condition by evaluating the mean intensity of the negative control specimens plus two standard deviations of those intensities in the equation for the best fit curve. The detection limits obtained in this manner were 1.7 pg r*Pf*CSP and 0.5 pg r*Pf*CSP for 0.1% T-20 and 0.5% T-20, respectively, at the 1:5,000 mAb dilution and 0.16 pg r*Pf*CSP and 0.5 pg r*Pf*CSP for the same conditions in the 1:20,000 mAb dilution. The lowest background and the stronger signal strength observed with the 0.1% T-20 buffer results in an overall greater sensitivity.

**Fig 3:**
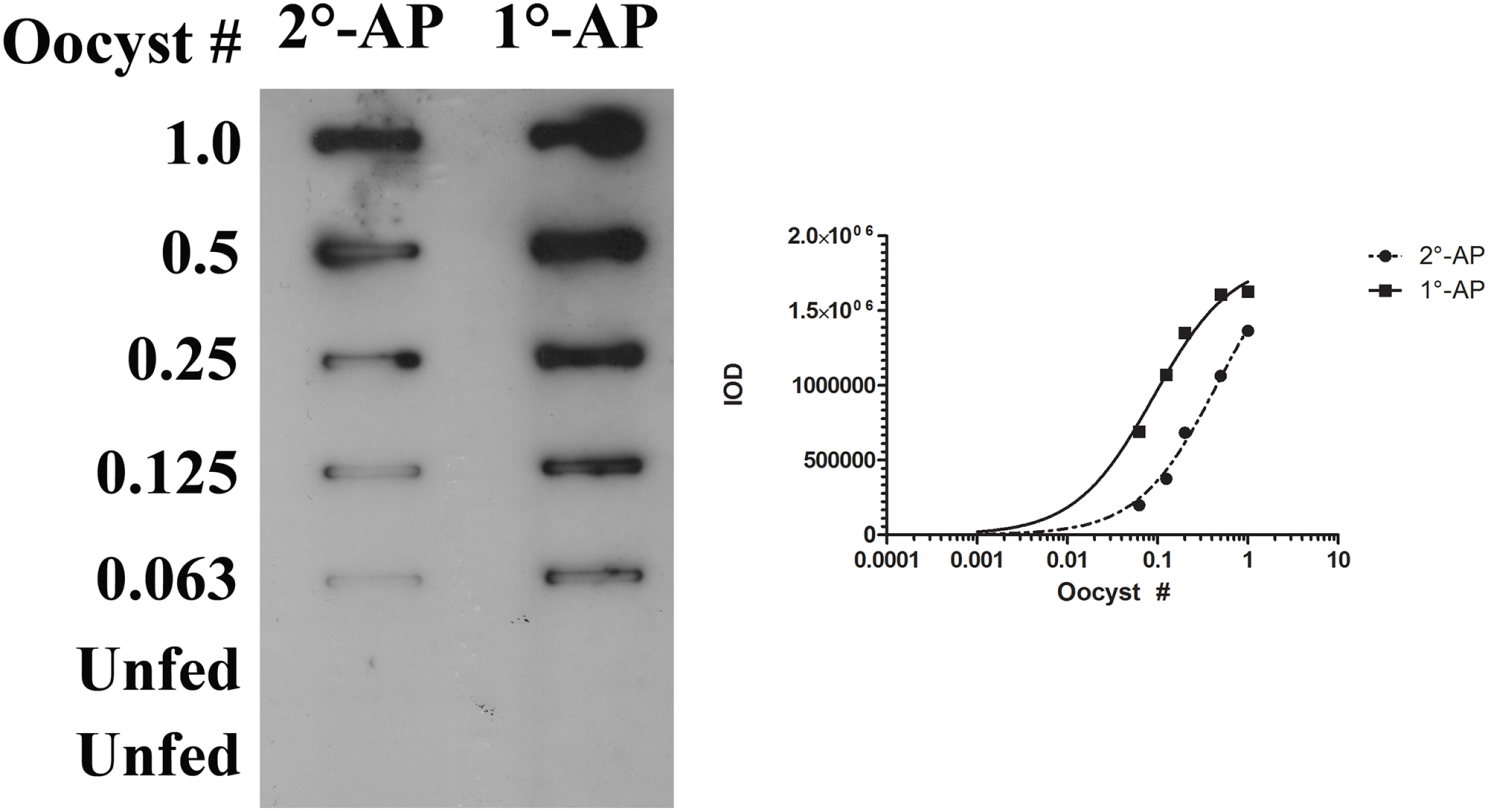
Labeling of primary antibody enhances detection of *Pf*Oocysts in the slot blot assay. We evaluated whether conjugation of primary mAb 2A10 with alkaline phosphatase (1°-AP) enhanced sensitivity for the detection of *Pf*oocyst prepared from mosquito lysates when compared to the use of AP-conjugated secondary antibody (2°-AP). Detection limits were compared for each protocol by fitting the band intensities of serially diluted oocysts to a Michaelis-Menten regression curve and establishing a cutoff intensity threshold of mean + 2 SD from unfed mosquito specimens run on the same blot. Labeled primary antibody displayed overall higher band intensities across the range of oocyst dilutions examined and achieved lower limits of detection than the typical sandwich antibody format (0.009 oocyst versus 0.02 oocyst, respectively). The removal of an additional antibody incubation step also contributed to an overall shorter assay time in the newly developed slot blot protocol.

To further improve the signal to noise ratio, we evaluated the possibility of eliminating a conjugated secondary antibody altogether in favor of a directly labeled primary antibody. Serial dilutions of oocyst lysate from whole mosquitoes were run on the slot blot assay along with unfed mosquito controls. Our results demonstrate that labeling of the primary mAb 2A10 enhanced signal intensity of low concentration samples, thus improving the sensitivity of the ECL SB significantly (Fig 3). These differences are less distinguishable at 1 oocyst per well where the intensity values obtained with both methods converge. This result is somewhat counterintuitive in that the use of a secondary antibody typically generates signal amplification; however, in the case of the slot blot assay the stronger signal observed with labeled primary antibody may be attributed to a number of possible causes: potentially higher affinity of the primary antibody for abundant NANP repeats than the secondary antibody at these low dilutions; potential loss of bound antibody and signal due to the increased wash steps required by a secondary antibody protocol. Briefly, the range of integrated optical densities was 199357–1365278 au for the standard conjugated secondary antibody and 691947–1628418 au for the labeled primary antibody. Further, the detection limit of a traditional sandwich antibody blot format results in a limit of detection of approximately 0.02 oocyst while labeling the primary antibody directly allows detection of as little as 0.009 oocyst. We therefore chose to utilize a labeled primary antibody in all future iterations of the slot blot assay.

### Performance of novel PfCSP mAb C3103 in slot blot

**Fig 4:**
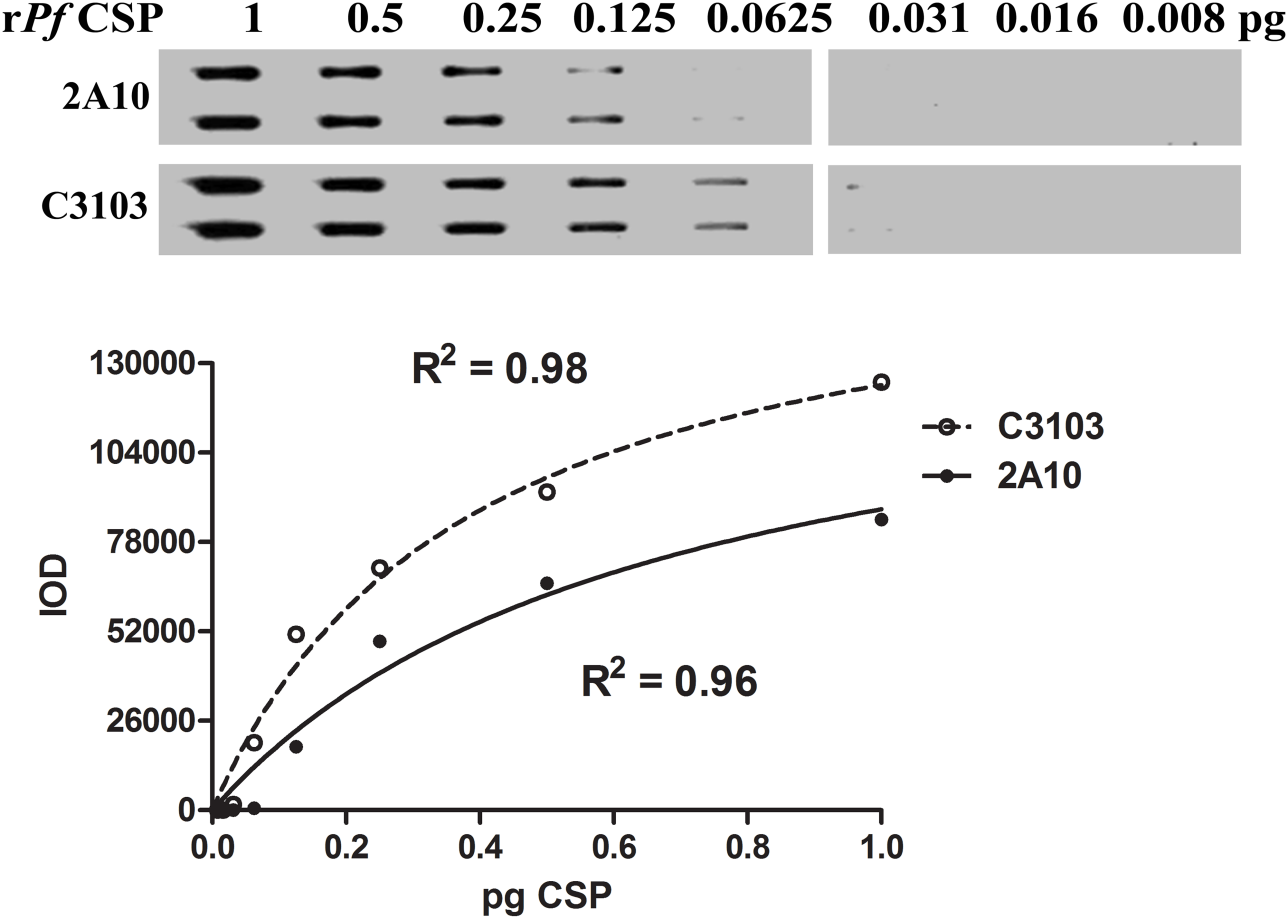
Sensitivity comparison between 2A10 and C3103 mAbs. Twofold serial dilutions of r*Pf*CSP protein were transferred to a nitrocellulose membrane using the slot blot apparatus and incubated with AP-conjugated 2A10 or C3103 mAb. Chemiluminescent signal was captured using autoradiography film and band intensities were quantitated using ImageJ software. While both antibodies adhere very well to the Michaelis-Menten curve fit, the C3103 antibody developed in our laboratory exhibited approximately 2-fold enhanced detection of CSP antigen.

In immunoassays, the limit of detection relies on the affinity and avidity of the antibody used to detect the target antigen. In order to increase the repertoire of available anti-*Pf*CSP mAbs, we have generated and characterized a new monoclonal antibody, mAb C3103, that recognizes the NANP-repeat unit epitope on *Pf*CSP. We compared the ability of the commonly used mAb 2A10 and mAb C3103 to detect both recombinant *Pf*CSP and parasite-derived *Pf*CSP in the slot blot assay. The mAb C3103 displayed significantly higher signal intensities over the entire range of serially diluted r*Pf*CSP concentrations tested (1–0.008 pg) (Fig 4). IOD values for mAb 2A10 ranged from 0–84518 au and had a mean intensity of 27333 au while IODs of the mAb C3103 ranged from 0–124523 au and had a mean intensity of 45009 au. Similarly, the C3103 antibody had a 2 fold lower limit of detection compared to the 2A10 mAb, detecting as little as 0.0625 pg of recombinant *Pf*CSP. Comparison of the mAb C3103 and mAb 2A10 against *Pf*CSP from oocysts in whole mosquito lysate displayed no significant differences in sensitivity (https://doi.org/10.6084/m9.figshare.4786039.v1).

### No film data capture using the ECL slot blot

**Fig 5:**
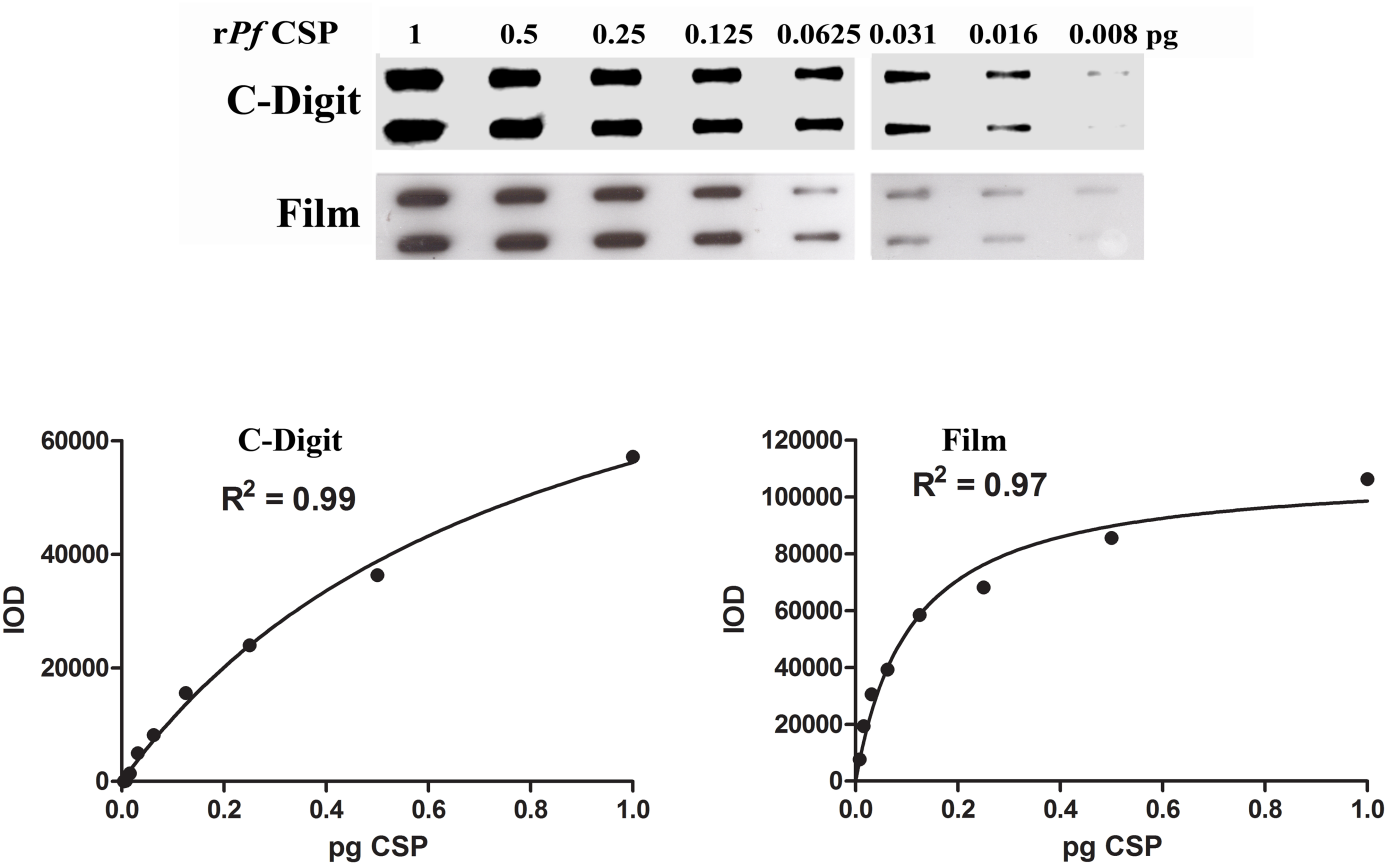
Comparison of film and digital scan data capture methods in the detection of r*Pf*CSP. Twofold serial dilutions of r*Pf*CSP protein were transferred to a nitrocellulose membrane using the slot blot apparatus and incubated with AP-conjugated C3103 mAb. Chemiluminescent signal from the membrane was captured using both autoradiography film and the C-Digit scanner. The resulting band intensities were fit to Michaelis-Menten regression curves to identify changes in the detection limit of the slot blot assay. Cutoff threshold intensities were determined from the mean intensity of negative control samples plus two standard deviations Film and the digital scan displayed similar sensitivities of approximately 0.01 pg of CSP. Intensities obtained on the C-digit scanner were generally bolder at lower intensities but declined more dramatically, with a steeper slope near the detection limit.

We next wanted to obviate the need for the AR film for data capture and analysis as the processing and development of AR films presents a number of logistical hurdles particularly in resource-limited field settings. In addition to necessitating access to specialized reagents and chemicals and performance of a somewhat tedious procedure, effective data capture onto AR films requires the use of a dark room. Furthermore, intensity resolution of a specific antigen-generated signal acquired on AR films necessitates minimizing nonspecific film exposure, which may not be an available option when sensitivity is low. Additionally, the use of a highly expensive charge-coupled device camera unit to capture digital exposures of the chemiluminescent signal is typically not feasible due to the high cost and maintenance of such equipment. We thus evaluated the performance characteristics of the slot blot assay when chemiluminescent signals on the membranes were analyzed via the LI-COR C-Digit Blot Scanner. The C-Digit represents an easy-to-use, relatively low cost blot scanner with a small footprint that could easily be deployed at multiple field sites to process ECL-SB testing results. In order to ensure no significant loss of sensitivity and detection capabilities would occur upon changing to digital capture of band intensities, single blots of serially diluted r*Pf*CSP protein and *Pf*oocysts were analyzed sequentially via both autoradiography film and the C-Digit scanner. Data acquired from both methods displayed similar levels of sensitivity and adhered very closely to the Michaelis-Menten Model. The C-Digit blot scanner could detect as few as 0.01 pg of r*Pf*CSP whereas analysis of developed films achieves a similar detection limit of 0.009 pg (Fig 5). While analysis of integrated optical densities from developed film usually provides higher overall numerical intensities (7699–106299 au) than digital data capture (0–57175 au), there are also certain limitations of film analysis. For example random variations in film exposure and development could contribute to a broad distribution of background intensities across the film. These variations allow residual, nonzero signal even in unfed mosquito specimens. In contrast, background intensities obtained via the C-Digit blot scanner are substantially more uniform and thus typically disappear upon background subtraction. Additionally detectable signal with the C-Digit blot scanner declines much more rapidly and suddenly than the slow and gradual disappearance of bands observed on developed films.

**Fig 6:**
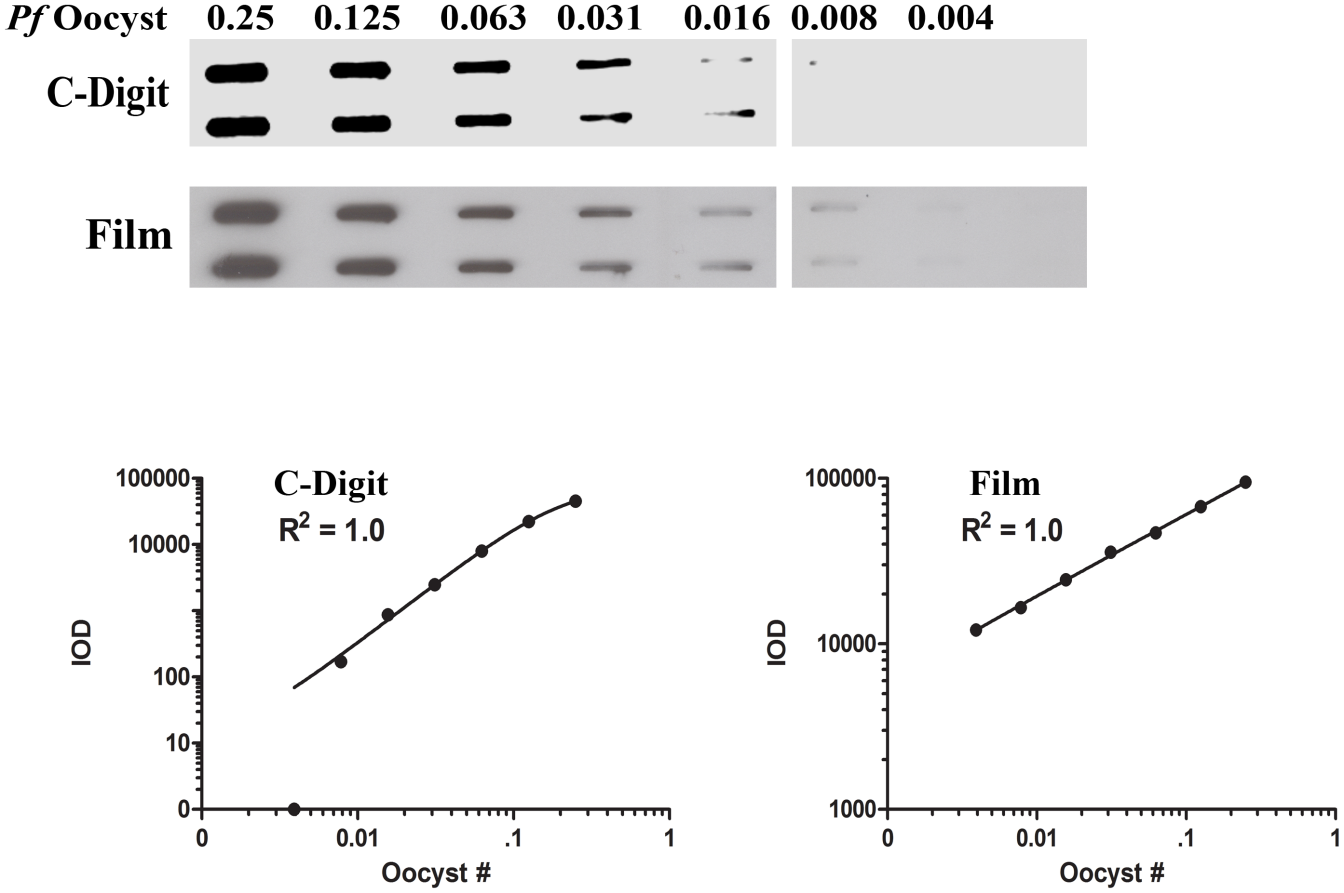
Comparison of film and digital scan data capture methods in the detection of *Pf* oocyst. Stock oocyst lysate generated from pooled infected mosquitoes was serially diluted and processed according to the ECL-SB assay protocol. Chemiluminescent signal from the membrane was obtained from both exposure to film and a digital scan. The resulting band intensities were plotted and detection limits were determined. Data were plotted on logarithmic scales to better visualize overall trends as linear scale axes results in four of the lowest concentration data points acquired on the LICOR residing closer to the horizontal Y = 0 axis than the higher concentration samples. Film displayed slightly better sensitivity for *Pf*oocysts, detecting as few as 0.01 oocyst whereas the C-Digit scanner could only detect as little as 0.02 oocyst. Both methods could demonstrate reactivity for infected mosquitoes with a single developing oocyst.

To determine whether the C-Digit scanner was a viable tool for the detection of *Pf* oocysts in whole mosquito lysates, we created serial dilutions of oocyst stock lysate prepared from pooled mosquitoes fed a *P*. *falciparum*-infected blood meal and again compared performance of the C-Digit scanner to traditional exposure of autoradiography film. Development of slot blot results using traditional film exposure results in approximately 2-fold higher sensitivity against *Pf* oocyst with a detection limit of 0.01 *Pf* oocyst for film (12895–90160 au) and 0.02 *Pf* oocyst for the C-Digit scanner (0–22325 au) (Fig 6). Interestingly, the sensitivity difference observed between both analysis methods is more prominent with oocyst samples than recombinant protein. This could be due to the fact that oocyst lysate is a more complex mixture of host proteins, other *Pf* proteins, and miscellaneous material are adsorbed to the slot blot membrane. Detection of *Pf*CSP antigen in lower dilutions of such samples is more difficult with potential for lower intensity bands which may not be adequately captured by the C-Digit blot scanner. Importantly however, both of these data acquisition methods are sufficiently sensitive to enable the detection of a single oocyst in the midgut of an infected mosquito.

### Reproducibility and assay precision

**Fig 7:**
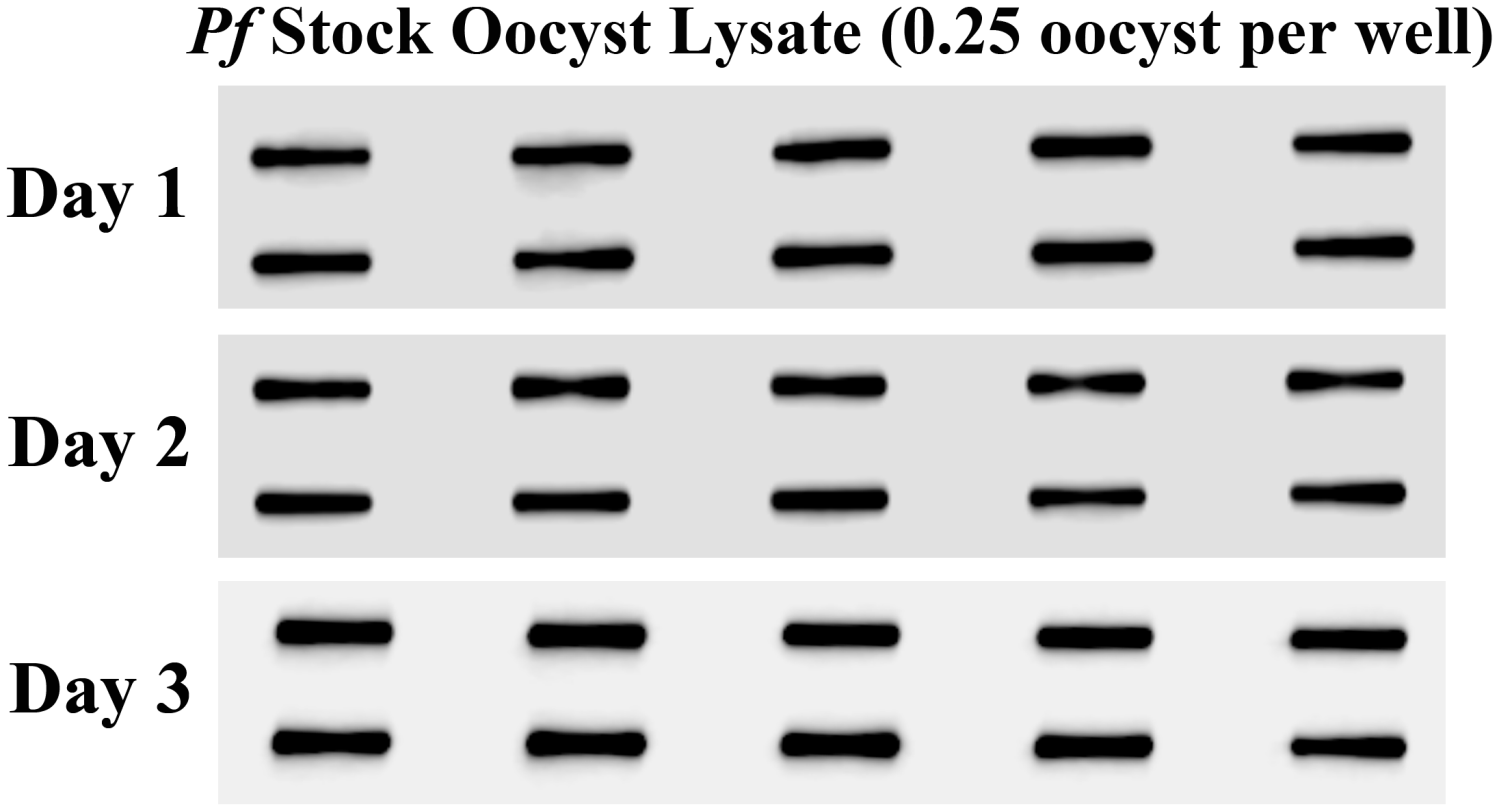
Inter-/Intra-assay varibility for the C-Digit blot scanner. In three separate experiments performed on three different days, 0.25 oocyst was loaded into each slot blot well and data was captured with the C-digit blot scanner. Variability in band intensities from each day never exceeded 18% and overall inter-assay variability was estimated to be 9.85%.

We confirmed the ability of the C-Digit blot scanner to precisely read and collect data. Ten replicates of an aliquot of *Pf* oocyst stock lysate (0.25 oocyst per well, a lower bound of oocyst burden that may be present in a test sample from endemic settings) were run simultaneously in three separate experiments on three separate days (Fig 7). Band intensities were measured using the C-Digit scanner software and inter- and intra-assay variation was determined as described in the Methods section. The data collected demonstrated that the means of all ten samples were fairly consistent from run to run of the assay, displaying a CV of 9.85% (Table 1). Within each run, individual samples displayed higher variability of 12–15% (Table 1), however, these values are in close agreement with the values observed using film [12].

**Table 1:** rPfCSP detection—Inter-/Intra-assay variability of 10 samples (S1-S10) over three days.

	IOD*												
Days	S1	S2	S3	S4	S5	S6	S7	S8	S9	S10	Mean	SD	CV (%)
**1**	18812	24421	23905	31652	24150	26040	23387	28099	32372	27149	25999	4042	15.55
**2**	26996	30191	27099	26403	28766	31430	25315	21022	21845	28426	26749	3331	12.45
**3**	35962	35746	33106	28978	22452	32800	36357	34501	29942	21395	31097	5438	17.49
**Grand Mean**											27948		
**SD**_**Means**_											2752		
**CV (%)											9.85		

* Integrated optical density was measured for 0.25 *Pf* Oocyst.

** CV (%) = (SD of Means/Grand Mean) x 100

### Prevalence estimation from whole mosquito lysates

**Fig 8:**
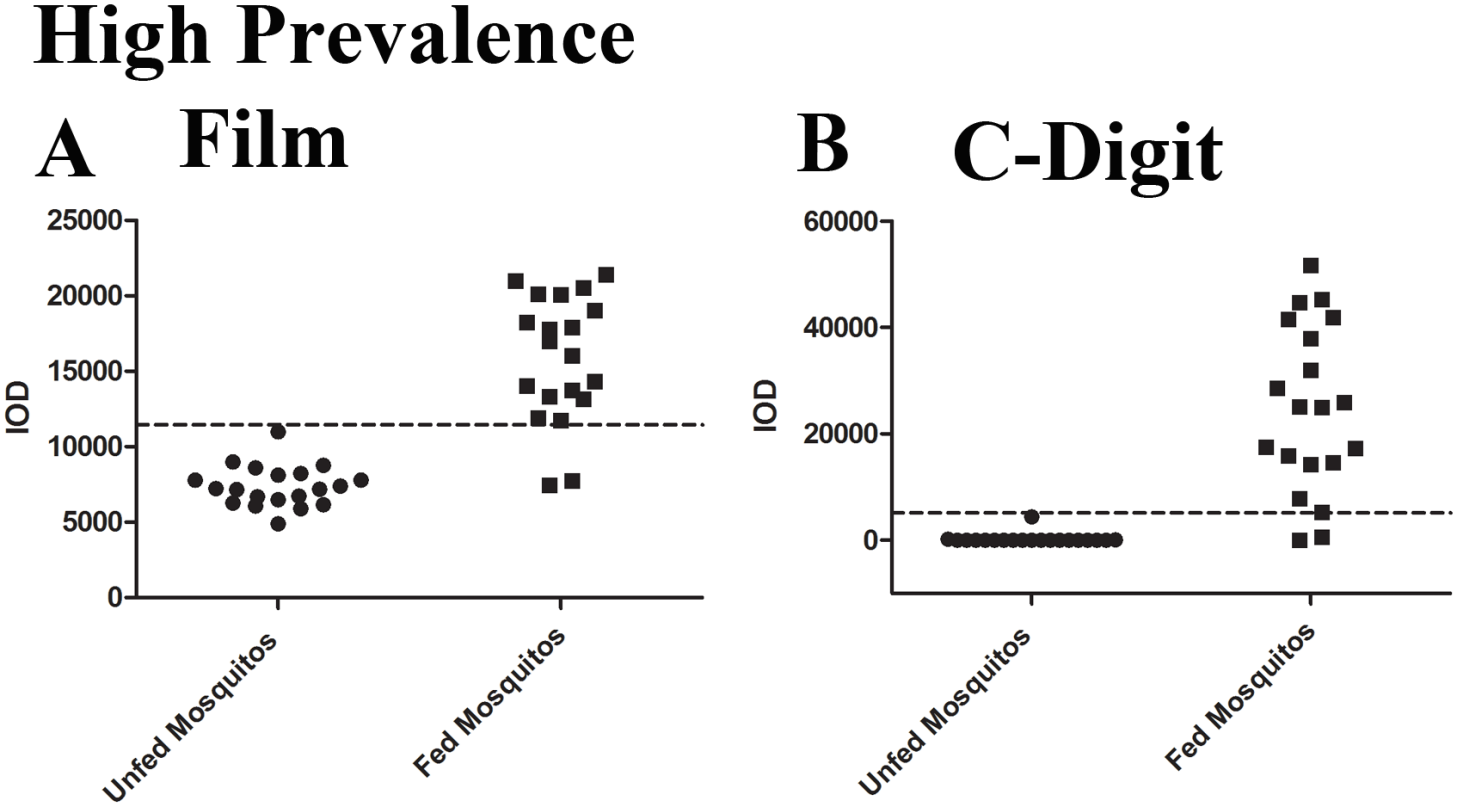
Prevalence estimation in high infectivity mosquito population using the C-Digit blot scanner. A batch of mosquitoes were fed an infectious blood meal with high percent *Pf* gametocytemia. On day 8 post feeding, a random sampling of 15 mosquitoes was dissected, stained, and analyzed using microscopy. Oocyst prevalence and intensity were estimated to be 86.7% and 20.5 oocysts per mosquito, respectively. This estimate was compared to the prevalence calculated from 20 different mosquitoes of the same batch that were homogenized and analyzed via slot blot data acquired using both film and the C-Digit blot scanner for data capture. Data obtained from both the film and blot scanner agreed very well, with 18/20 mosquitoes testing positive (90% prevalence) in both methods. The cutoff threshold for each analysis was determined by calculating the mean + 2SD of the band intensities of the negative or unfed mosquitoes.

**Fig 9:**
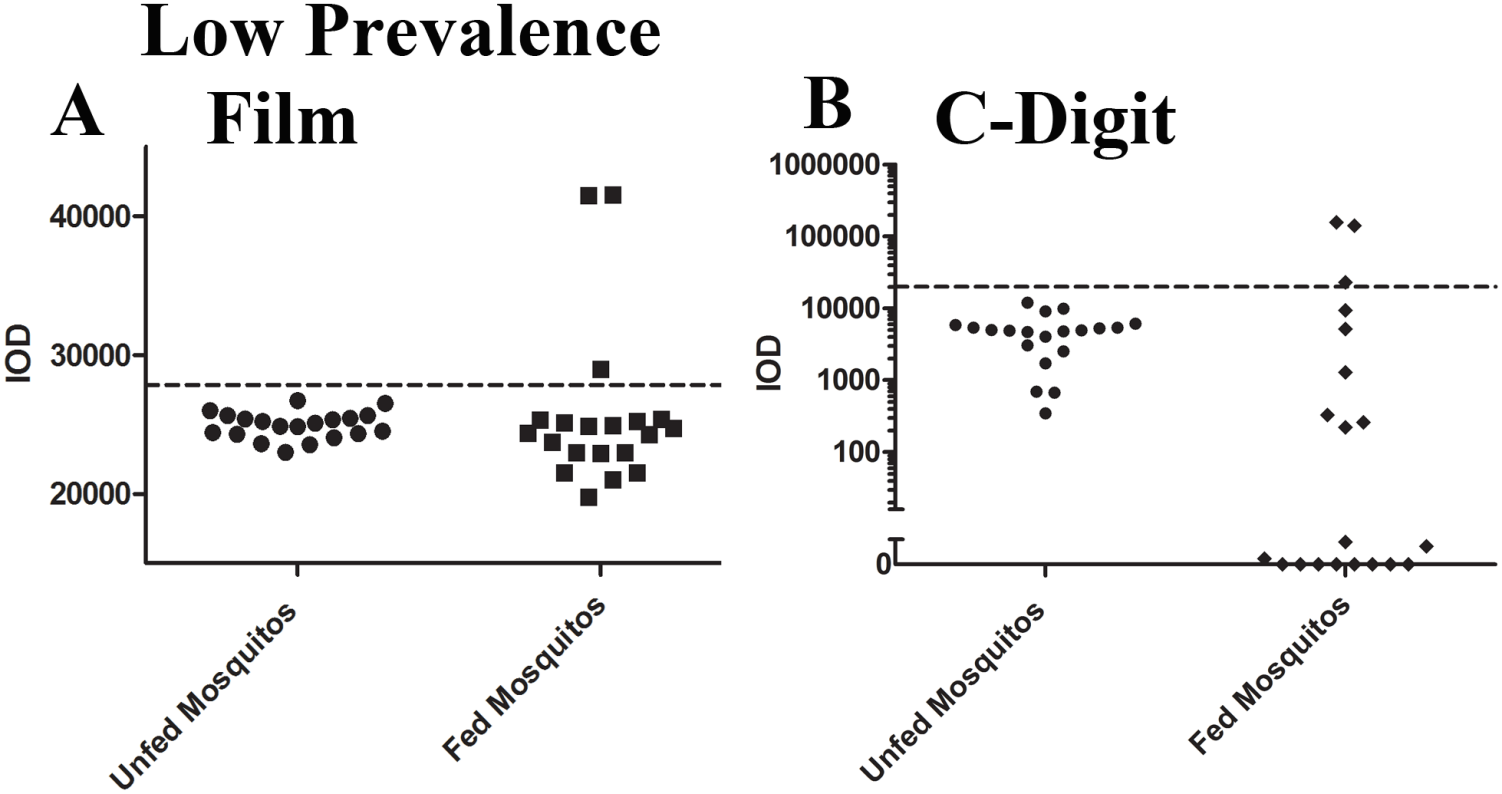
Prevalence estimation in low infectivity mosquito population using the C-Digit blot scanner. A batch of mosquitoes were fed an infectious blood meal with high percent *Pf* gametocytemia. On day 8 post feeding, a random sampling of 15 mosquitoes were dissected, stained, and analyzed using microscopy. Oocyst prevalence and intensity were estimated to be 20% and 1.4 oocyst per mosquito, respectively. This estimate was compared to the prevalence calculated from mosquitoes of the same batch that were homogenized and analyzed via slot blot data acquired using both film and the C-Digit blot scanner for data capture. Data obtained from both the film and blot scanner agreed very well, with 3/20 mosquitoes testing positive (15% prevalence) in both methods.

To establish that no significant loss of sensitivity occurs as a result of the transition to completely digital image capture and analysis, we compared *P*. *falciparum* prevalence estimations from one high burden (with a geometric mean oocyst count of 20.5 oocysts/mosquito) and one low burden (with a geometric mean oocyst count of 1.4 oocyst/mosquito) infected mosquito population utilizing both film and C-Digit capture methods. Samples were processed according to the slot blot protocol and then individual membranes were analyzed both by exposure to autoradiography film and by image capture on the C-Digit scanner. The cut-off thresholds for determination of positive samples was determined as described elsewhere in this manuscript. Briefly, samples with intensities greater than the mean intensities of negative or unfed mosquito specimens plus 2 standard deviations were identified as positive. In the high oocyst burden population, film detection and analysis of band intensities identified 18/20 mosquitoes as positive for a prevalence of 90% (Fig 8A). This agrees very closely with the estimate obtained from microscopy of 86.7% (13/15 mosquitoes identified as positive). The C-digit scanner likewise identified 18/20 positive mosquitoes and 90% prevalence demonstrating that no loss of information occurs when the data is captured and analyzed digitally (Fig 8B). The χ^2^ test between these two methods, which yield the same results, establishes a p value of 1.0. Importantly, these observations are only estimates of the population prevalence; our intent was primarily to demonstrate that the C-Digit blot scanner and the slot blot assay did not offer completely divergent assessments of infected mosquito counts. The intensity distributions between the C-Digit and the slot blot generally agree well with a number of distinct specimens definitively identifiable as positive. Importantly, uninfected mosquitoes analyzed on the C-Digit scanner displayed negligible levels of nonspecific background signal. This is in direct contrast with results from film analysis where fundamental variations inherent to the film development process can contribute to a broader range of intensity values even in negative specimens. In the low oocyst burden population, signal intensities obtained from autoradiography film were relatively consistent with only 3/20 mosquitoes identified as positive for a prevalence of 15.0% (Fig 9A). The C-digit scanner similarly obtained a prevalence estimate of 15.0% with only 3/20 mosquitoes testing positive (Fig 9B). The cutoff threshold here was established as before, with the calculated mean intensity of unfed samples plus two standard deviations. One mosquito identified as positive by both film and the C-Digit scanner, exhibited a band intensity close to the cutoff threshold for both methods, potentially leading to an equivocal status determination. However since both methods yielded a positive test result in a sample which is close to the cut-off value, this result suggests that C-digit and autoradiography would be equally effective in detecting borderline low positive samples.

We noted that some infected mosquitoes in the C-Digit dataset had lower IOD values than the unfed mosquito group (Fig 9B). Since the IOD values in infected mosquitoes were significantly higher, the C-Digit was still able to differentiate between a positive and negative test result. Overall, we find that the data acquired from the C-digit and autoradiography are consistent and there is no substantial difference in these estimates and those obtained from microscopy of 20% prevalence (3/15 mosquitoes identified as positive) in this particular mosquito population with a χ^2^ p value of 1.0.

Our data establish that the modified slot blot is a sensitive, easy-to-use assay for the detection of target antigen with broad applicability not only in the development and characterization of vaccine products but also as a potential screening tool for multiple different pathogens. The protocol as outlined here is easily adaptable for the specific detection of infectious agents for which a well characterized biomarker and appropriate antibody exists. With the overall reduction in assay time and the establishment of a “no film” procedure for the acquisition and analysis of data, the slot blot also has a relatively low technological cost that enables widespread adoption of the procedure in potentially resource-limited environments. However, the benefits of this assay would be more broadly applicable for large epidemiological and vaccine studies where thousands of samples may need to be processed in a short duration. In contrast to microscopy, this assay can be extensively validated for sensitivity, specificity, and precision studies. Importantly, data can be permanently stored in digital format for analytical and statistical investigations and results can be made available for subsequent analyses. Future implementation of the assay at field sites in endemic areas as well as potential optimization and validation of the multiplex detection of additional biomarkers to enhance early detection of developing parasites will fully realize the slot blot’s potential as a screening assay.
